# *In silico* Structural, Functional and Phylogenetic Analyses of cellulase from *Ruminococcus albus*

**DOI:** 10.1186/s43141-021-00162-x

**Published:** 2021-04-19

**Authors:** Anila Hoda, Myqerem Tafaj, Enkelejda Sallaku

**Affiliations:** Department of Animal Sciences, Faculty of Agriculture and Environment, Agricultural University of Tirana, Koder Kamez, 1029 Tirana, Albania

**Keywords:** *In silico* analysis, Physicochemical properties, Rampage, Phylogenetic relationships, Structure alignment

## Abstract

**Background:**

Cellulose is the primary component of the plant cell wall and an important source of energy for the ruminant and microbial protein synthesis in the rumen. Cell wall content is digested by anaerobic fermentation activity mainly of bacteria belonging to species *Fibrobacter succinogenes, Ruminicoccus albus, Ruminococcus flavefaciens, and Butyrivibrio fibrisolvens*. Bacteria belonging to the species *Ruminococcus albus* contain cellulosomes that enable it to adhere to and digest cellulose, and its genome encodes cellulases and hemicellulases.

This study aimed to perform an *in silico* comparative characterization and functional analysis of cellulase from *Ruminococcus albus* to explore physicochemical properties and to estimate primary, secondary, and tertiary structure using various bio-computational tools.

The protein sequences of cellulases belonging to 6 different *Ruminococcus albus* strains were retrieved using UniProt. In *in silico* composition of amino acids, basic physicochemical characteristics were analyzed using ProtParam and Protscale. Multiple sequence alignment of retrieved sequences was performed using Clustal Omega and the phylogenetic tree was constructed using Mega X software. Bioinformatics tools are used to better understand and determine the 3D structure of cellulase. The predicted model was refined by ModRefiner. Structure alignment between the best-predicted model and the template is applied to evaluate the similarity between structures.

**Results:**

In this study are demonstrated several physicochemical characteristics of the cellulase enzyme. The instability index values indicate that the proteins are highly stable. Proteins are dominated by random coils and alpha helixes. The aliphatic index was higher than 71 providing information that the proteins are highly thermostable. No transmembrane domain was found in the protein, and the enzyme is extracellular and moderately acidic. The best tertiary structure model of the enzyme was obtained by the use of Raptor X, which was refined by ModRefiner. Raptor X suggested the 6Q1I_A as one of the best homologous templates for the predicted 3D protein structure. Ramachandran plot analysis showed that 90.1% of amino acid residues are within the most favored regions.

**Conclusions:**

This study provides for the first time insights about the physicochemical properties, structure, and function of cellulase, from *Ruminococcus albus*, that will help for detection and identification of such enzyme *in vivo* or *in silico*.

**Supplementary Information:**

The online version contains supplementary material available at 10.1186/s43141-021-00162-x.

## Background

Cellulases are hydrolytic enzymes that hydrolyze ß-1,4-glycosidic linkage within cellulose. The complete hydrolysis of cellulose is obtained by the action of three types of cellulases namely endoglucanase, exoglucanase, and ß-glucosidase. Cellulose is the primary component of the plant cell wall and an important source of energy for the ruminant and microbial protein synthesis in the rumen. The importance of fiber digestion is increasing, especially in the development of feeding strategies for ruminants. The cell wall contents are digested in both the liquid and solid phases of the rumen contents by anaerobic fermentation mainly through rumen bacteria. According to Henderson et al. [1], *Prevotella, Butyrivibrio, Ruminococcus,* and other unclassified members of Lachnospiraceae, Ruminococcaceae, Bacteroidales, and Clostridiales accounted for 67.1% in a pool of bacterial sequence data collected from different ruminant species fed different diets. These might be considered a “core bacterial microbiome”.

The cultivable bacteria mostly involved in fiber digestion are *Fibrobacter succinogenes, Ruminicoccus albus, Ruminococcus flavefaciens, and Butyrifibrio fibrisolvens* [2]. Bacteria belonging to the species *Ruminococcus albus* contain cellulosomes that enable them to adhere to and digest cellulose, and its genome encodes cellulases and hemicellulases [3]. *Ruminococcus albus* is a primary cellulose degrader that produces acetate usable by its bovine host. The complete genome of this bacteria is fully described by Suen et al. [3]. Densities of the rumen fiber-digested bacterial species, including *Ruminococcus albus* were influenced by different feed-related factors (concentrate level, fiber quality, and particle size, among others) [4–6], as well as animal-related factors [6].

Very little is known about the structure of cellulases. Islam and Roy (2018) [7] have isolated and characterized by morphological and biochemical analysis the cellulases from *Paenibacillus* sp., *Bacillus* sp., and *Aeromonas* sp. of 3D protein structures. Experimental determination of 3D protein structures is very difficult and complex [8], also expensive and time-consuming; therefore, other approaches have to be considered [9]. In this context, bioinformatics tools are of great interest and are widely applied for the prediction of 3D protein structure in several cases [8, 10–12], or gene analysis [13]. Cellulase from genus *Bacillus* is previously *in silico* analyzed [14]. The aim of the present study is the characterization by the use of bioinformatics tools of enzyme cellulase from *Ruminococcus albus* not previously investigated. The present study envisaged the computational prediction of the secondary and tertiary structures of cellulase (P23660), structure evaluation, and the functional characterization including protein–protein interaction.

## Methods

### Sequence retrieval, alignment and phylogenetic analysis

Cellulase protein sequence from *Ruminococcus albus* was retrieved in FASTA format (accession no. P23660) from UniProt (Universal Protein Resource (https://www.uniprot.org/)) and served as a query for BLAST at http://blast.ncbi.nlm.nih.gov/Blast.cgi against a non-redundant protein database. Clustal Omega (version 1.2.4) algorithm was used for the alignment of retrieved protein sequences through multiple sequence alignment.

The same sequence was used as a query sequence for the PSI-BLAST against protein data bank (PDB) at http://blast.ncbi.nlm.nih.gov/Blast.cgi to identify its homologous structures. PRALINE at http://www.ibi.vu.nl/programs/pralinewww/ was used for the alignment of query and template sequences.

Phylogenetic tree of all the total 6 bacterial cellulase protein sequences from different *Ruminococcus albus* strains has been constructed through the maximum likelihood method based on JTT matrix-based model [15] by the use of MEGAX [16] software. The reliability of internal branches was assessed by using 1000 bootstrap replicates, and gaps were detected in the analysis.

### Primary sequence analysis and subcellular localization

The online software, Protparam [17] at http://expasy.org/tools/protparam.html was used for the determination of physicochemical properties of selected sequences such as amino acid composition, aliphatic index (AI), isoelectric point (pI), instability index (II), number of positive and negative charged residues, grand average of hydropathicity (GRAVY), and extinction coefficient (EC).

CELLO subcellular localization predictor at http://cello.life.nctu.edu.tw/ [18], TMHMM server v. 2.0 [19], and PSLpred, a SVM-based method for the subcellular localization of prokaryotic proteins at http://crdd.osdd.net/raghava/pslpred were employed to predict the subcellular position.

### Secondary structure, topology, and signal peptide prediction

To predict the secondary structure of the protein, two online server SOPMA at https://npsa-prabi.ibcp.fr/cgi-bin/npsa_automat.pl?page=npsa_sopma.html and PSIPRED v3.3 (http://bioinf.cs.ucl.ac.uk/psipred/) [20] were applied, and results obtained from these tools were also compared to determine α-helix, ß-sheet, turns, and loops.

TopCons [21] (http://topcons.cbr.su.se/) predicts consensus topology of membrane proteins and signal peptides (SPs). Signal P 4.1 server [22]at http://www.cbs.dtu.dk/services/SignalP/ searches for the presence of signal peptide cleavage sites.

### 3D structure prediction using homology modeling, model evaluation, and refinement

The full 3D structure of cellulase from *Ruminococcus albus* is not available in the Protein Data Bank (PDB). Therefore, we used five online homology modeling programs to generate a 3D structural model for cellulase, using the FASTA format of the query sequence (P23660). The tertiary structure of query sequence was predicted through these programs: Expasy SWISS-MODEL (ProMod Version 3.70), Phyre2 [23] (http://www.sbg.bio.ic.ac.uk/phyre2/html/page.cgi?id=index), RaptorX structure prediction server, (http://raptorx.uchicago.edu/StructurePrediction/predict/), (PS)^2^-V2 [24] (http://ps2.life.nctu.edu.tw/), and LOMETS (Local Meta-Threading-Server) [25] which is a protein structure prediction server at http://zhang lab.ccmb.med.umichedu/LOMET S/).

The ModRefiner (http://zhanglab.ccmb.med.umich.edu/ModRefiner/), which is a high-resolution protein structure refiner, was used to improve the physical quality of structures. The built and refined models were evaluated via Rampage at http://mordred.bioc.cam.acukrapper/rampage.php. The Ramachandran plots were depicted for each model. The model with the least number of residues in the disallowed region was selected for further studies. The model in specified format was submitted to Protein Data Bank.

### Structure alignment

The best predicted 3D structure of the protein was structurally aligned and compared with the selected template structure from PDB. The alignment was done by the use of Dali server (http://ekhidna.biocenter.helsinki.fi/dali/) [26], by superposition of the atomic coordinate sets and a minimal root mean square deviation (RMSD) between the structures. The RMSD of two aligned structures indicates their divergence from one another.

### Functionally analysis

For functional analysis CYS_REC tool (http://linux1.softberry.com/berry.phtml) was used to identify the position of cysteine and compute the most probable SS bond pattern of pairs in protein [27]. The set of conserved amino acid residues were analyzed using Motif search tool (http://www.genome.jp/tools/motif/). COFACTOR at http://zhang lab.ccmb.med.umich.edu/ COFACTOR/ predicts the biological function of proteins based on their structure, sequence, and protein–protein interaction (PPI).

Identification of protein–protein interaction was carried out by STRING 11.0 (https://string-db.org/) [28] which is used to construct a protein–protein interaction network for different known and predicted protein interactions.

Pocket regions are defined by the use of several online servers, GHECOM (Grid-based HECOMi finder) server at http://strcomp.protein.osaka-u.ac.jp/ghecom/ and CastP server (http://sts.bioe.uic.edu/castp/). Depth (http://mspc.bii.a-star.edu.sg/tankp/help.html) was used for predicting depth, cavity sizes, ligand binding sites, and PKA.

## Results

### Sequence retrieval, alignment and phylogenetic analysis

The amino acid sequence of the cellulase enzyme (P23660) was retrieved from UniProt database in FASTA format. This sequence served as the query for BLAST and six cellulase sequences were obtained, with the similarity of at least 84%, belonging to different strains of *Ruminococcus albus* (Table 1). The total number of amino acid residues ranged from 364 to 414, with molecular weights that lie between 41,218 to 45,880 Da. They belong to endoglucanase and ß-glucanase. The cellulases show the different catalytic mechanisms of the endohydrolysis of (1 to > 4)-beta-D-glucosidic linkages in cellulose, lichenin and cereal beta-D-glucans and endohydrolysis of (1 to > 4)-beta-D-xylosidic linkages in xylans.

**Table 1:** Characterization of retrieved sequences of cellulases for different *R. albus* strains using UniProt tool

No.	Organism	Accession number	Protein	Number of aa	Molecular weight (Da)	Molecular function	Family
1.	*Ruminococcus albus*	P23660	Endoglucanase A	364	41,218	Glycosidase, Hydrolase	GH5
2.	*Ruminococcus albus*	A0A011UFY5	Endoglucanase	413	45,742	Glycosidase,	GH5
3.	*Ruminococcus albus*	A0A1H7KSB4	Endoglucanase	413	45,676	Glycosidase,	GH5
4.	*Ruminococcus albus*	A0A1I1KHA3	Endoglucanase	413	45,815	Glycosidase,	GH5
5.	*Ruminococcus albus*	Q59733	Beta-1,4-D-glucanase	414	45,880	Glycosidase, Hydrolase	GH5
6.	*Ruminococcus albus*	E6UGI8	Cellulase	410	45,776	Glycosidase, Hydrolase	GH5

A BLASTp search against Protein Data Bank (PDB) was carried out, to find the most suitable protein structures as templates. The results of the BLASTp are displayed in Table 2, which shows the first 10 hits with the highest scores. The query coverage is higher than 93%, and the percentage of identity ranged from 35.15 to 44.96%.

**Table 2:** The first 10 hits with the highest scores of BLASTp on the cellulase sequence against Protein Data Bank (PDB)

No.	Accession number	Max score	Total score	Query coverage	*E* value	Per. ident	Accession length	Resolution(Ǻ*)*	Description
1.	1EDG_A	249	249	98%	2e–79	38.46%	380	1.6	*Ruminiclostridium cellulolyticum*
2.	6MQ4_A	294	294	95%	1e–97	40.61%	353	1.4	*Hungateiclostridium cellulolyticum*
3.	4NF7_A	217	217	94%	2e–67	35.15%	363	2.11	*Butyrivibrio proteoclasticus*
4.	4IM4_A	293	293	93%	4e–97	43.77%	336	2.42	*Hungateiclostridium thermocellum*
5.	6GL2_A	268	268	93%	1e–87	40.29%	337	1.96	*Zobellia galactanivorans*
6	6WQP_A	303	303	93%	5e–101	43.71%	354	1.6	*Ruminococcus champanellensis*
7.	3NDY_A	283	283	93%	2e–93	43.31%	345	2.1	*Clostridium cellulovorans*
8.	3AYR_A	303	303	93%	1e–100	44.96%	376	2	*Piromyces rhizinflatus*
9.	3AYS_A	301	301	93%	9e–100	44.67%	376	2.2	*Piromyces rhizinflatus*
10.	6Q1I_A	291	291	93%	4e–96	43.87%	357	1.35	*Clostridium longisporum*

Figure S1 shows the multiple sequence alignment for cellulases from different strains of *Ruminococcus albus*, obtained by Clustal Omega software. All sequences were highly conserved, with absolute conservation regions (*) and relative (.) conservation regions. Also, query sequence (P2360) and 10 template sequences are aligned and the results of homology between them are shown in Figure S2.

**Fig. 1:**
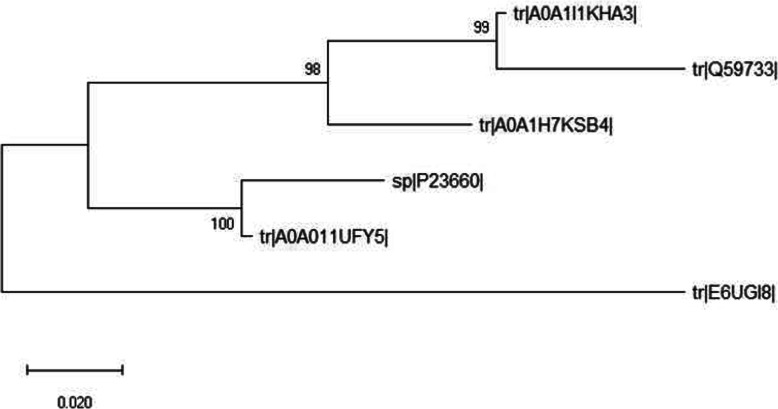
Phylogenetic tree generated via Mega X software through maximum likelihood method based on JTT matrix-based model, showing the evolutionary relationship among cellulase sequences from different *Ruminococcus albus* strains. The bootstrap consensus tree is inferred from 1000 replicates, with the confidence values shown next to the branches

The phylogenetic tree of amino acid sequences from different strains of *Ruminococcus albus* is shown in Fig. 1. It has been constructed with MEGA X, using maximum likelihood method based on the JTT matrix-based model. The bootstrap values at the node are higher than 90%, indicating the robustness of the tree. There are two major groups present and one outgroup. The horizontal branches represent evolutionary lineages.

### Primary sequence analysis and subcellular localization

The physicochemical properties details, like isoelectric point (pI), extinction coefficient, instability index (II), aliphatic index (AI), and Grand Average of Hydropathicity (GRAVY) for selected enzymes from different *Ruminococcus albus* strains are given in Table 3.

**Table 3:** Physicochemical properties of selected proteins, from different strains of *Ruminococcus albus*

No.	Accession no.	Theoretical pI	Extincion coefficient	Instability index	Aliphatic index	GRAVY
1.	P23660	4.53	95465	25.73	73.96	− 0.601
2.	A0A011UFY5	4.39	95465	29.13	71.58	− 0.594
3.	A0A1H7KSB4	4.40	97080	26.53	72.18	− 0.585
4.	A0A1I1KHA3	4.37	95590	28.89	73.21	− 0.552
5.	Q59733	4.44	90090	28.85	73.28	− 0.556
6.	E6UGI8	4.39	92610	33.92	78.55	− 0.649

All sequences have similar values of the isoelectric point that lies between 4 and 4.5 which indicated the moderate acidic nature of the proteins. The extinction coefficients (EC) showed slight variation between cellulases of all strains. The values of instability index for all selected sequences were less than 40, indicating that the proteins are stable. The results indicated that Ai values ranged between 71.58 and 78.55, which means that the proteins are thermostable. The GRAVY value represents the protein–water interactions. The GRAVY values were found to be negative and ranged between − 0.552 and − 0.649, indicating the hydrophilic nature of the enzyme.

Possible disulfide linkages in the primary sequences are given in Table 3. In most of the cases, disulfide bridges were present.

**Fig. 2:**
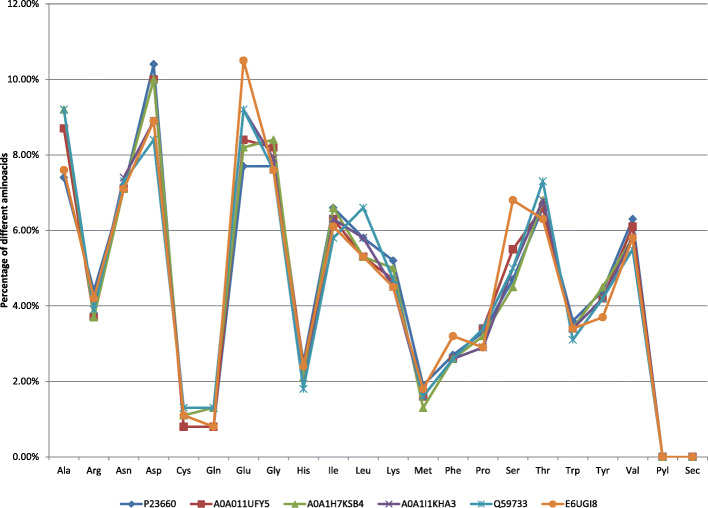
Graphical representation of the amino acid composition of selected cellulase sequences

A comparison of amino acid composition of cellulases from six strains of *Ruminococcus albus* is shown in Fig. 2. The X-axis represents the amino acid composition, while the Y-axis represents the percentage of each amino acid residue, while the color bars represent selected sequences.

The subcellular position of cellulase from *Ruminococcus albus* is predicted by different tools. CELLO predicted that the enzyme was extracellular with the highest reliability of 0.864. Also, PSLpred predicted the protein sequence as extracellular with a reliability index of 3.294. The subcellular position of cellulase from *Ruminococcus albus* is predicted by using TMHMM Server, v.2.0. Summary outputs revealed that the enzyme has no transmembrane helix (Figure S3).

### Secondary structure topology and signal peptide prediction

**Fig. 3:**
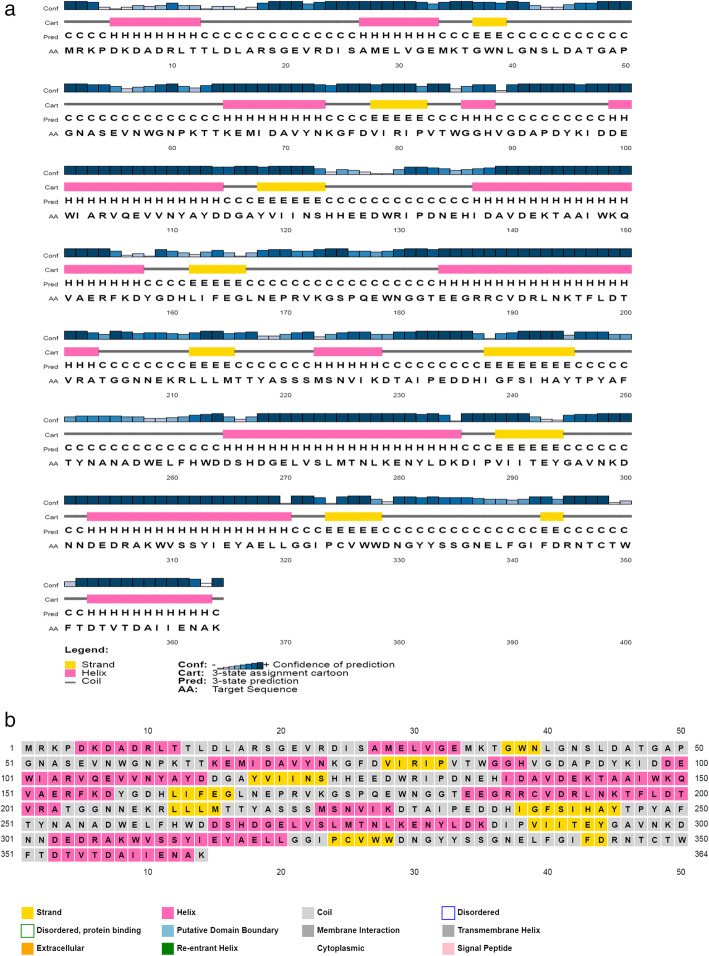
Secondary structure map (**a**) and graphical representation of the predicted secondary structures present within the protein P23660 (**b**) attained by PSIPRED (The pink blocks represent the alpha helices; the yellow blocks represent beta strands, and the black thread-like structures were the coils. The confidence of prediction observed throughout the predicted secondary structure was quite high, indicating high reliability of the prediction

The secondary structure of selected cellulase sequences was estimated using SOPMA tools. The percentage of alpha helix, extended strand, beta turn, and random coils in these sequences from different *Ruminococcus albus* and from 10 template sequences are shown in Table 4. From these results, it is observed that random coils are dominant in all sequences, followed by alpha helix and extended strand. The query sequence displayed the lowest percentage of random coils (42.31) compared with other sequences and the highest value of beta turn (5.77). The secondary structure map and a graphical representation of query sequence (P23660) predicted by PSIPRED [20] are shown in Fig. 3a and b. A graphical presentation of query and template secondary structure alignment is shown in Figure S4.

**Table 4:** Predicted secondary structure content and disulfide bridges from 6 cellulase proteins of *Ruminococcus albus* strains and from 10 selected template structures

No.	Accession no.	Alpha helix	Extended strand	Beta turn	Random coil	Disulfide bridge prediction
Cellulase protein						
1.	P23660 (Target)	37.91	14.01	5.77	42.31	None
2.	A0A011UFY5	37.05	12.11	4.60	46.25	23–398
3.	A0A1H7KSB4	36.80	12.59	4.84	45.76	23–362
4.	A0A1I1KHA3	40.44	12.11	4.36	43.10	23–398
5.	Q59733	40.34	12.08	4.11	43.48	23–398
6.	E6UGI8	38.78	13.41	4.88	42.93	23–375
Templates						
7.	1EDG_A	35.00	12.63	5.00	47.37	None
8.	6MQ4_A	35.98	15.01	5.10	43.91	None
9.	4NF7_A	37.47	13.50	4.68	44.35	117–221
10.	4IM4_A	37.80	15.18	3.87	43.15	None
11.	6GL2_A	36.20	15.43	4.45	43.92	235–304
12.	6WQP_A	35.59	14.12	4.24	46.05	207–217
13.	3NDY_A	36.81	13.91	4.93	44.35	None
14.	3AYR_A	39.89	13.03	5.05	42.02	None
15.	3AYS_A	35.11	15.69	6.12	43.09	41–221
16.	6Q1I_A	36.41	14.01	4.76	44.82	None
17.	1EDG_A	35.00	12.63	5.00	47.37	None

TMHMM and TOPCONS revealed that the protein has no transmembrane helixes and is present outside the membrane part of the cell (Figure S3). SignalP suggests no signal peptide.

### 3D Structure prediction using homology modeling, model evaluation, and refinement

The 3D models of cellulase from *Ruminococcus albus* (P23660) were gained by different protein structure homology model building programs: SWISS-MODEL Homology Modelling, Raptor X, PS^2^-V2, Phyre 2, and Lomets.

Phyre2 suggested the 1EDG_A template as one of the best homologous templates for a possible 3D cellulase protein structure, with 100% confidence and 97% coverage. The same template was suggested also by PS^2^V2, with alignment at 98%, *e* value 2.6e–18, and 37.93% identity. Submission of cellulase to the Swiss Model server generated one protein structure model, where the best template was 3AYS_A showing 44.67% sequence identity, resolution 2.20, sequence similarity 0.43, and coverage 0.93. The best model predicted by Lomets was generated using 3AYR_A as a template with 1550 Norm Zscore. RaptorX suggested 6Q1I_A as the best template for the 3D cellulase structure, with a *p* value 2.22e–09. All models obtained by these programs were refined by ModRefiner, to refine the protein structure closer to the native.

**Fig. 4:**
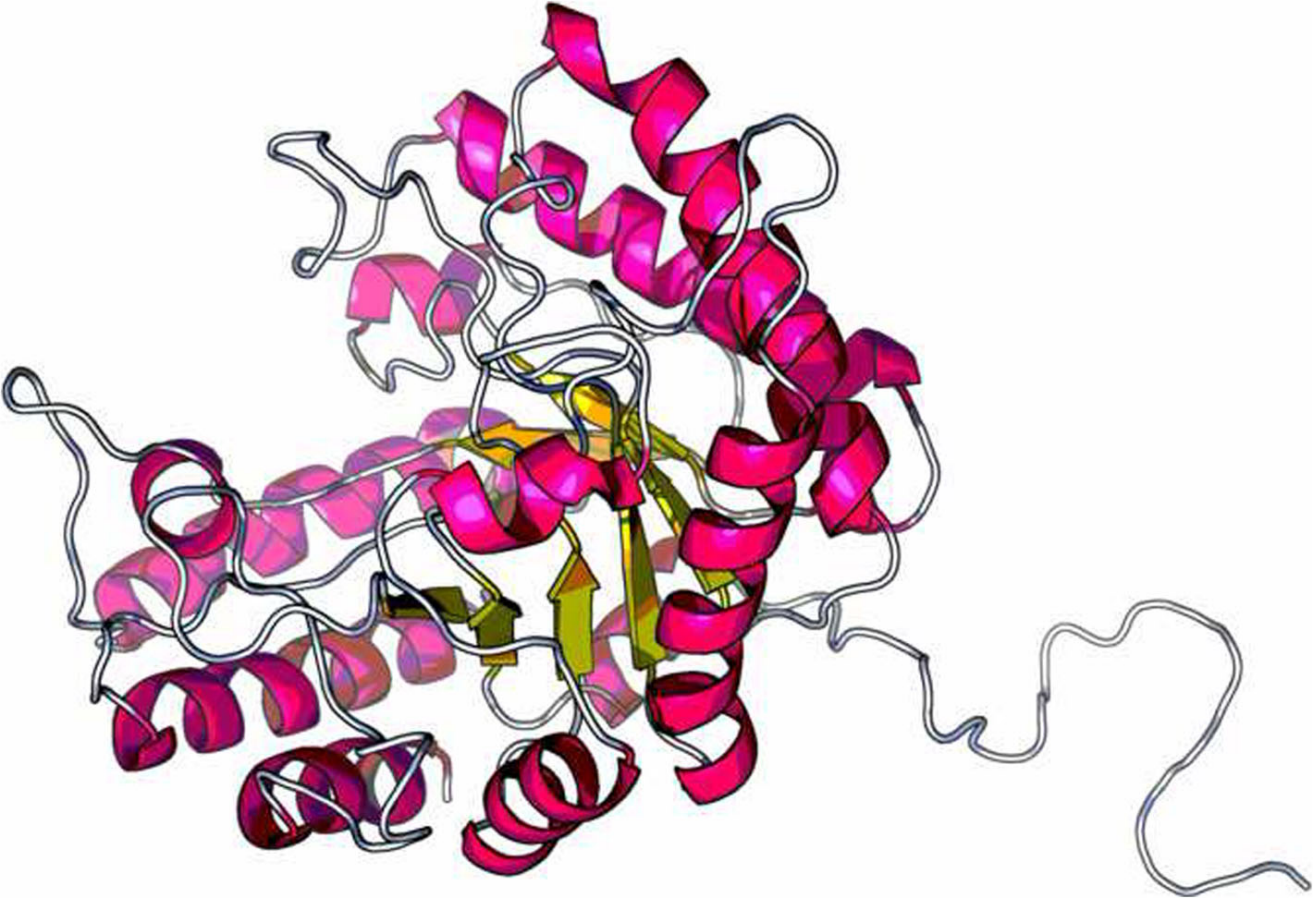
Predicted 3D structure of cellulase from *Ruminococcus albus* provided by Raptor X and refined by ModRefiner

The initial and refined models were taken for validation analyses by PROCHECK [29]. RAMPAGE validates 3D models by plotting the Ramachandran plot. In the Ramachandran plot of all models, the percent residues were located in favored, allowed, and disallowed. The Ramachandran plot of each model is compared, and the results are shown in Table 5. The best model was generated with Raptor X, with PDB ID: 6Q1I, as a template. The percentage of favored regions is 88.5% and with the minimum percent of the disallowed region (0); meanwhile, the refined model of Raptor X (Fig. 4) showed the highest percentage of the favored region (90.1), which implies the characteristics of a good quality model.

**Table 5:** The Ramachandran plot structure validation of original and refined structures

Model		Favored region (%)	Additional allowed region (%)	Generously allowed region (%)	Disallowed region (%)
Swiss model	Original	86.4	12.6	0.7	0.3
	Refined	87.7	11.3	0.3	0.7
Raptor X	Original	88.5	10.2	1.2	0.0
	Refined	90.1	9.0	0.6	0.3
PS^2^-V2	Original	85.4	12.7	1.6	0.3
	Refined	90.4	7.8	0.6	1.2
LOMETS	Original	83.5	13.0	2.5	0.9
	Refined	90.1	7.8	1.2	0.9
Phyre	Original	82.6	12.7	2.5	2.2
	Refined	88.8	9.3	1.2	0.6

### Structure alignments

**Fig. 5:**
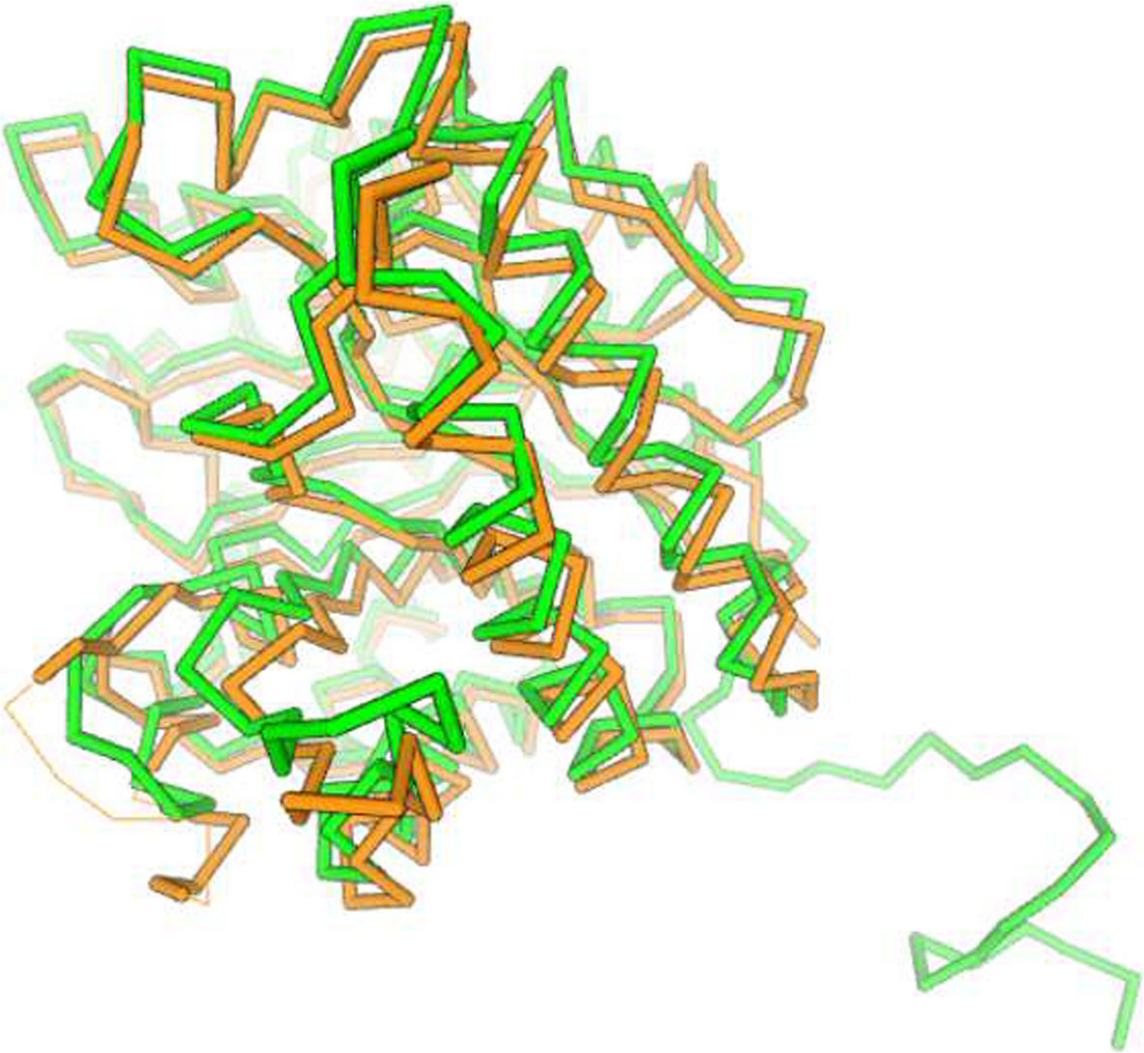
Dali 3D structure alignment between query sequence P23660 (green) and template 6Q1I (brown)

The final refined 3D protein structure model was superimposed with the structure of the template 6Q1I_A. The outputs are shown in Fig. 5 and indicate geometrical and structural similarity. The calculated z score was 53 and RMSD was 1.1. Most query and template structures are matched in tertiary structure alignment.

### Functional analysis

Two functional motifs were detected, which were found to be a member of the glycoside hydrolase family (Figure S5).

**Fig. 6:**
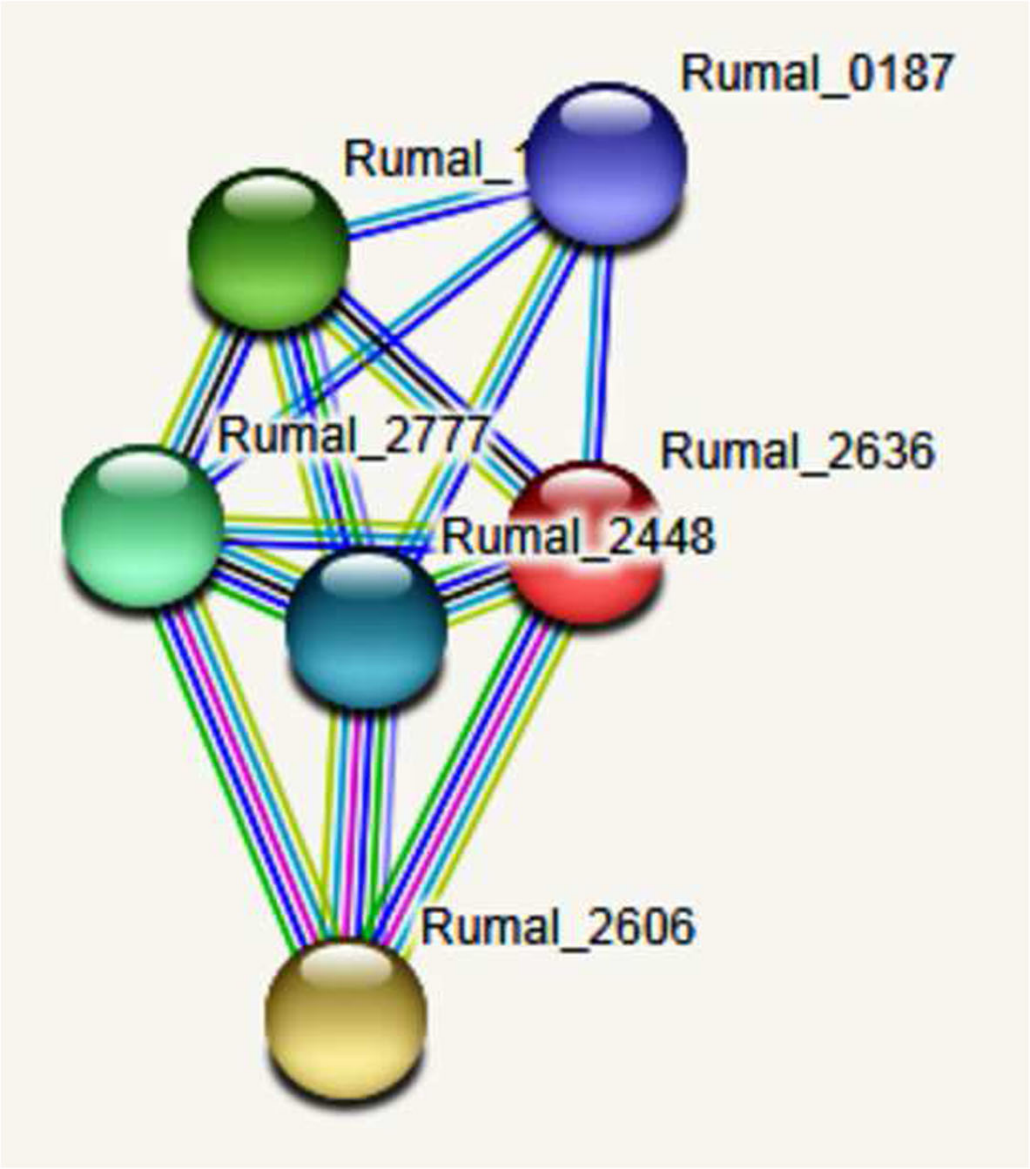
Protein–protein interaction map for the cellulase of *Ruminococcus albus*

Functional analysis revealed five potential interacting partners of cellulase in the protein interaction network as resolved by STRING analysis (Fig. 6). The query protein Rumal have five closest interacting protein with cellulase, endoglucanase, and glycosyltransferase activity. The STRING database analysis depicted that the protein–protein interaction (PPI) network is comprised of 6 nodes connected with 14 different edges. The expected number of edges was 6, while the average node degree score was 4.67 which means that one node had at least 4.67 interacting nodes. The average local clustering coefficient was 0.933 and PPI enrichment *p* value was observed as 0.00152. Protein–protein interaction (PPI) networks showed that cellulase interacted with 5 other proteins in a very high score of confidence. The closest interacting protein was Rumal_2606 (Cellulose 1.4 beta cellobiosidase), with the shortest node with a score of 0.973. It belongs to the glycoside hydrolase family protein. Then Rumal_1050 (Endoglucanase) had a score of 0.968 belonging to the glycoside hydrolase family 9. Rumal 2777 (Endoglucanase) is part of the glycoside hydrolase family 5 and had a score of 0.946, Rumal_2448 (cellulase) is part of the glycoside hydrolase family 9 and had a score of 0.943, and finally, Rumal_0187, with glycosyltransferase function had a score of 0.914.

**Fig. 7:**
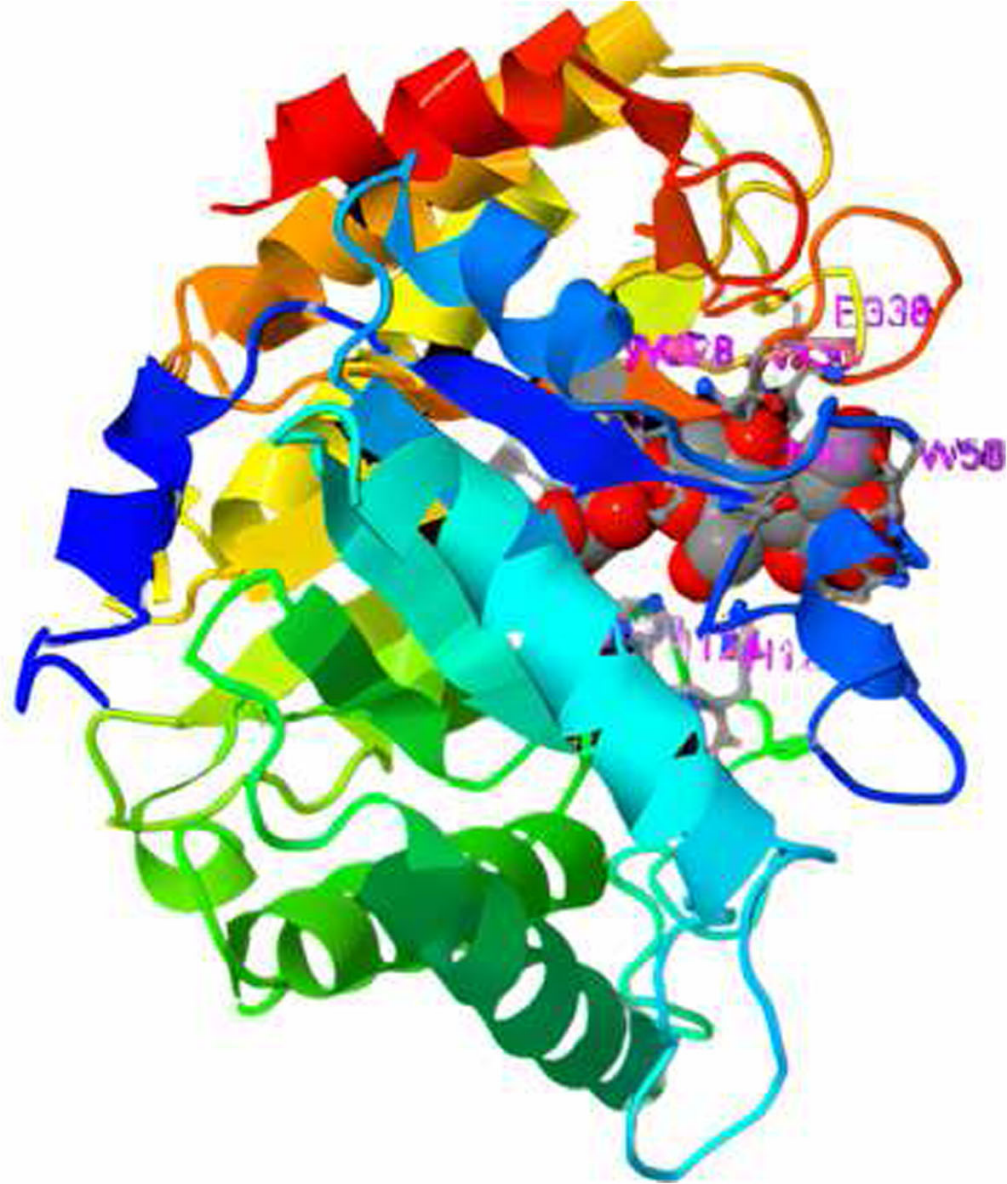
Predicted ligand binding sites of cellulase from *Ruminococcus albus*

Ligand binding sites determined by the use of COFACTOR software indicate the conserved residues with the highest Cscore, which is the confidence score of the predicted binding site. Cscore for the predicted binding site is 0.69. The residues in the predicted binding site are as follow: 42, 58, 124, 125, 168, 245, 293, 328, 330, and 338. BS-score which is a measure of local similarity (sequence & structure) between template binding site and predicted binding site in the query structure was 1.67 (BS-score > 1) representing a significant local match between the predicted and template binding site (Fig. 7).

**Fig. 8:**
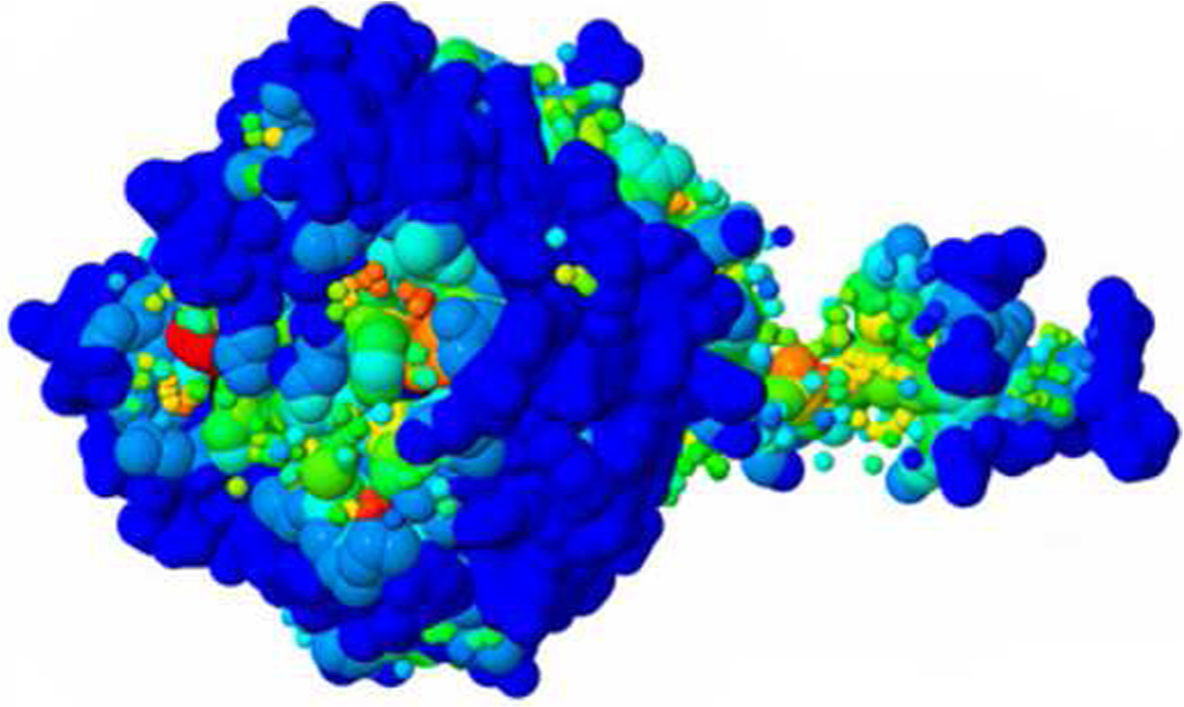
GHECOM results, Jmol view of pocket structure based on pocketness color

GHECOM server finds five pockets on protein surfaces using mathematical morphology, and the results of pocket structure based on pocketness color are shown in Fig. 8. The pockets contribute to the formation of binding sites and active sites of protein [30, 31].

**Fig. 9:**
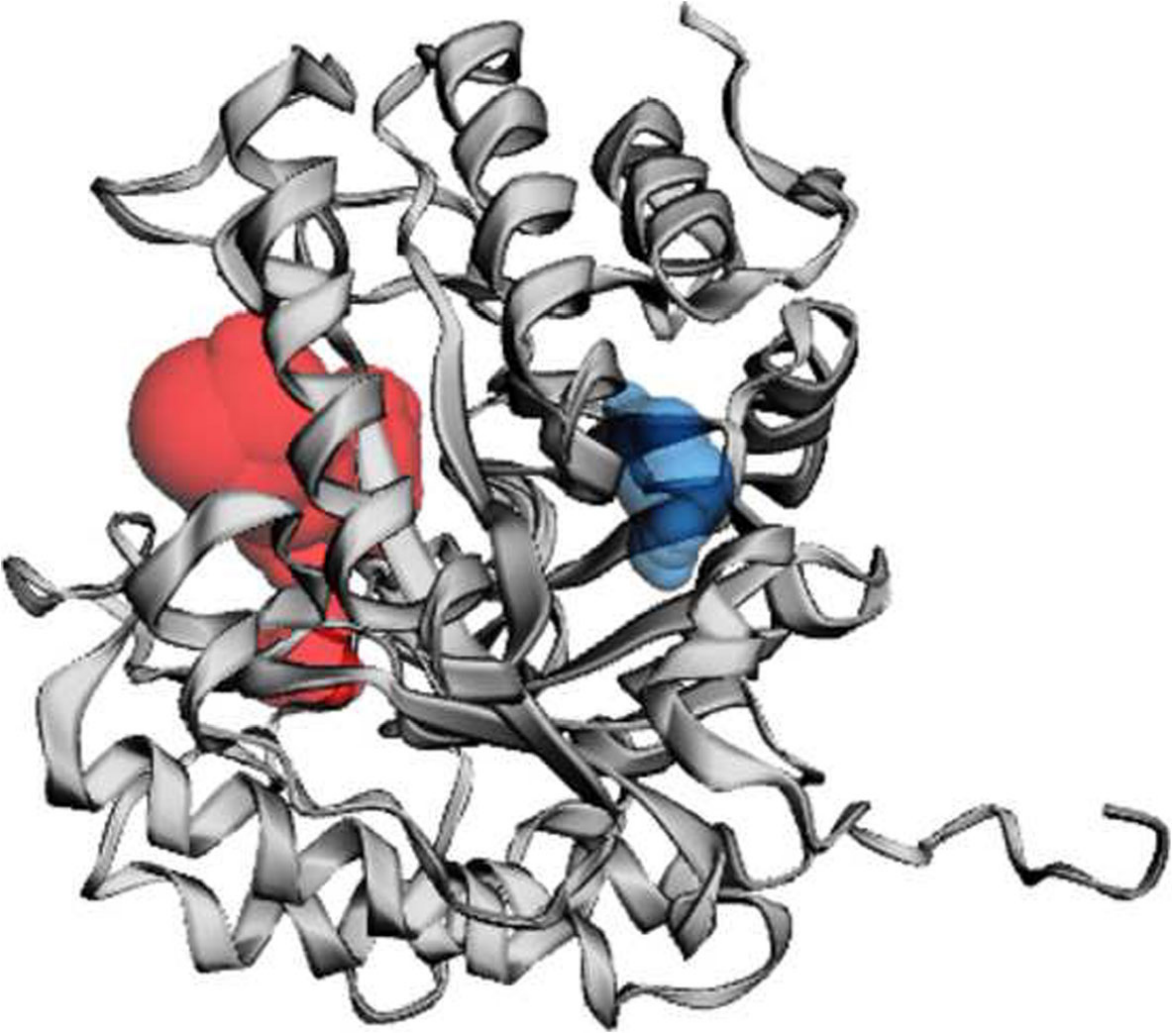
CastP results showing surface accessible pockets as well as interior inaccessible cavities

The pockets predicted by CASTp are shown in Fig. 9, where different cavities are shown in different colors, based on area and volume size; the most important is illustrated in red color. The largest pocket has an area of 370.310 and a volume of 324.097 amino acids. The second pocket has an area of 63.733 and a volume of 21.367 amino acids.

**Fig. 10:**
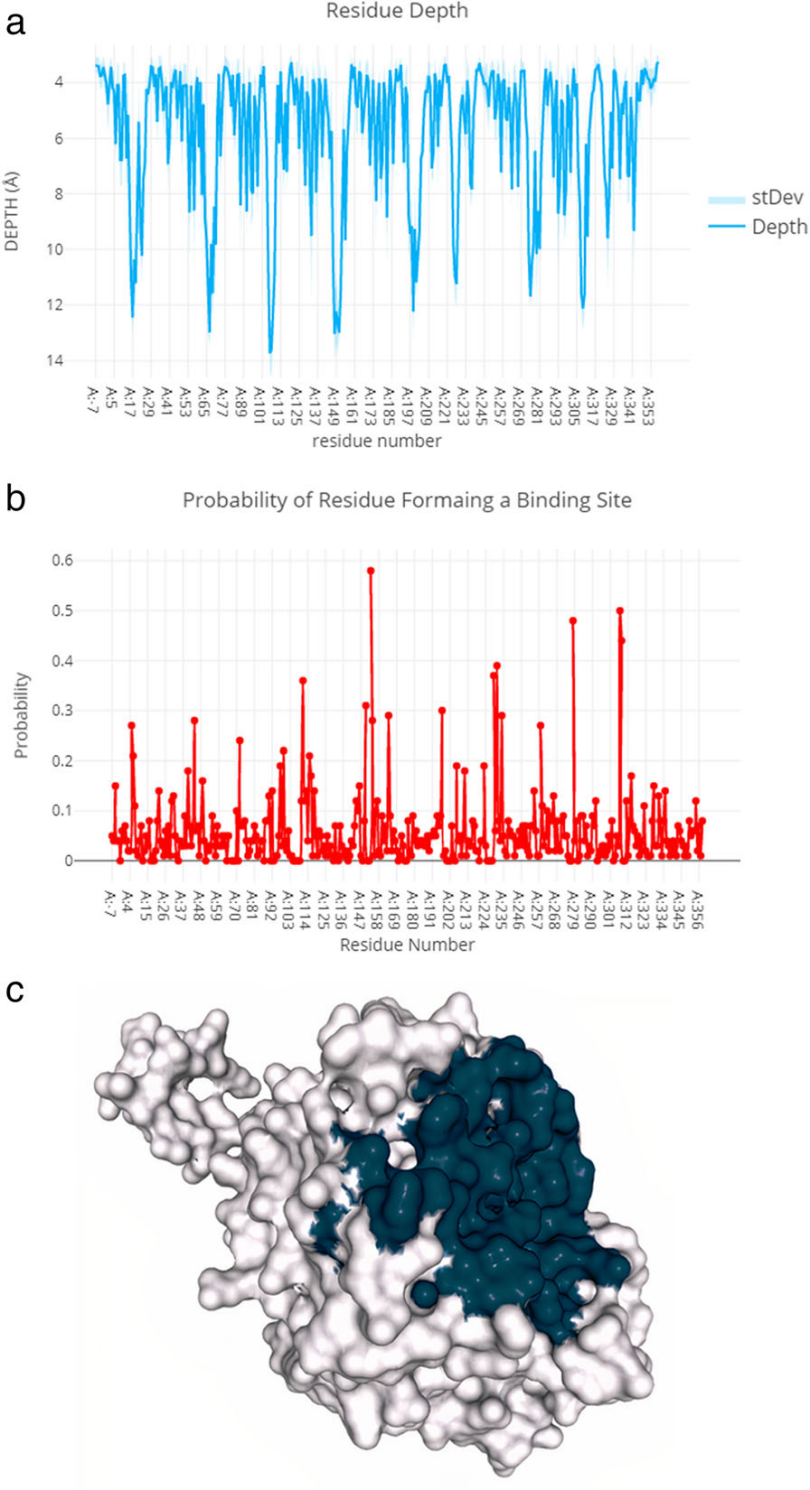
Residue depth plot (**a**), probability of residue forming a binding site (**b**), and a 3D rendition of the cavity prediction (**c**)

The probability of residue forming a binding site and residue depth plot and a 3D rendition of the cavity prediction is shown in Fig. 10.

## Discussion

Cellulases are complex enzymes that are produced by different organisms. Cellulases play an important role in different areas of industry and in animal feeding to enhance the digestibility of fiber–rich roughage fed to ruminants [32]. Two different cellulolytic enzymes from black goat rumen have been characterized [33, 34]. Also, an *in silico* analysis of cellulases from *Bacillus* sp. is previously done [14], but from *Ruminococcus albus* is not analyzed earlier in detail by bioinformatic tools.

According to Sefid et al. [31], the use of bioinformatics tools is a compelling strategy to close the gap between the number of protein sequences and the 3D protein structure. Computational tools are increasingly used to focus the search in sequence space, enhancing the efficiency of laboratory evolution [35]. Adyaman et al. [10] admit that in silico protein modeling is comparatively cheaper and faster than experimental determination methods.

Consequently, *in silico* analysis of protein structure is one of the very useful methods for studying the structural and functional aspects of the protein [8]. *In silico* analysis of proteins has played a great contribution recently in the field of computational biology illustrating the structural and functional aspects of proteins [36–39].

The present study has considered the phylogenetic, structural, and functional analysis of cellulase from *Ruminococcus albus*. The phylogeny of cellulases from 6 selected strains indicates that there are two groups in these strains. The tree is of high reliability since the bootstrap values are 98–100%.

This study has demonstrated several physicochemical characteristics which determine the uniqueness of a molecule. According to Mohanta et al. [40], the isoelectric or isoionic point of a protein is the pH at which a protein carries no net electrical charge and is considered neutral. Prediction of pI is essential in the development of buffer systems for purification and isoelectric focusing [41]. A protein is considered as alkaline in nature if the pI value is greater than 7, and acidic when the value is below 7. In this study, the pI values of all selected cellulase sequences ranged between 4.39 and 4.53 suggesting a moderately acidic nature of these cellulases, like in some *Bacillus* sp. [14], but the cellulases from *Bacillus subtilis* were alkaline [14]. The instability index (II) indicates protein stability. Proteins with II higher than 40 are referred as unstable [42]. The instability indices of all selected cellulase sequences from different *Ruminoccocus albus* were less than 40; therefore, the enzymes are considered stable. Also, the cellulases from different *Bacillus* sp. were found to be stable [14]. The aliphatic index is the relative volume of the protein occupied by the aliphatic amino acids in the side chain [43] and plays role in protein thermal stability. The values of aliphatic indices were more than 71, indicating a thermostable nature of all enzymes. This is in line with the fact that *Ruminococcus albus* is one of the few organisms that ferment cellulose to form ethanol at mesophilic temperatures *in vitro* [44]. The thermostable behavior of the protein is suitable for the dairy industry [37], in the sugar industries, where high temperatures are required for efficient extraction. The hydrophobic or hydrophilic character of cellulases is analyzed with the GRAVY score. GRAVY values were found to be negative, indicating that the proteins are nonpolar and hydrophilic. The acidic and stable nature of these enzymes allows them to survive in the moderate acidic environment of the rumen of ruminant species. The pH values 5.8 to 6.4 are considered as an optimal pH range for the activity of cellulolytic bacteria, including *R. albus*, and cellulases. If the pH value in the rumen felt below 5.5, the activity of cellulolytic and consequently fiber digestion is strongly reduced. On the contrary, in this pH range, the activity of mainly starch-fermenting taxa such as *Prevotella* is very high. On this basis, several authors (among them Zebeli et al. [5]) recommend that if the pH value remains below 5.8 for an interval of more than 5–6 h during the day, this is a sign of subacute ruminal acidosis in dairy cows (SARA).

The selected cellulase sequences from different strains of *Ruminococcus albus* have similar variations in amino acid compositions, as can be seen in Fig. 3. This composition implies a similar function and hydrolyses the same substrate. The prediction of protein secondary structures from sequences is considered as a bridge between the primary sequences and tertiary structure prediction [45]. Based on secondary structure prediction, it was observed that cellulases from all strains were classified in random coils and alpha helix. There was no disordered protein binding site present, and proteins are not unfolded. The high percentage of the alpha helix structure indicates that the enzymes are thermostable, which is in concordance with the high values of the aliphatic index. The cysteine residues are very important because they may take part in the formation of disulfide bonds between various parts of the protein. Disulfide bonds play an important role in folding and stabilizing the unfolded form of the protein by lowering the entropy [12]. Lugani et al. [14] found that alpha helix was dominant in *Bacillus pumilus,* whereas extended strand and the random coil were observed to be dominant in *Bacillus subtilis* and *Paenibacillus polymyxa.* The enzyme was extracellular, and this was supported also by the TMHMM tool which indicates no transmembrane domain present in the protein.

Prediction of 3D model of a protein by *in silico* analysis is a highly challenging aspect to corroborate the data obtained from the NMR or X-ray crystallography-based methods [37]. The query sequence (P23660) was blasted against PDB to find the best template. The selection criteria were lower *E* value and higher query coverage and maximum identity. The accuracy of the predicted model depends on the degree of sequence similarity. In our study, the query and template sequences shared 39.94–44.96% identity, which means that more than 80% of the C-atoms can be expected to be within 3.5 Å of their true position [9].

All models provided by different servers were evaluated. The best model of the tertiary structure was obtained by Raptor X where 6Q1I, which is endoglucanase from *Clostridium longisporum*, has been used as a structural template. Model refinement is important to improve the quality of predicted models. The refinement of the predicted 3D protein model is carried out by ModRefiner. It is a crucial step in bringing the models closer to experimental accuracy for further computational studies [10]. The Ramachandran plot for the predicted model showed that more than 90% of the residues are in the favored region, implying a good quality model [46].

The alignment of the predicted model and template structure (6Q1I) is applied by Dali software to evaluate the similarity between structures. The value of RMSD indicates the degree to which both three-dimensional structures are similar. The smaller the value of RMSD, the more similar the structures are. Thus, the predicted model was confirmed to be reliable and accurate. The results confirmed the reliability of the structure predicted by RaptorX. Therefore this theoretical structure was deposited at PMDB database and the accession number of the model is PM0083494.

## Conclusions

Cellulases are complex enzymes that are produced by many microorganisms including fungi and bacteria that degrade cellulose. There are a lot of areas of industry, including the animal feed industry and ruminant feeding, where microbial cellulases have a great application. Therefore *in silico* analysis of the physicochemical features of a protein is very important to get a theoretical overview of the enzyme. This study presents the first reported structural analysis of cellulases from *Ruminococcus albus*. Phylogenetic analysis was performed and indicated two major groups in cellulases from different *Ruminococcus albus* bacterial strains. From this study, it was found that cellulase is an extracellular, acidic, hydrophilic, and thermostable enzyme with a molecular weight of about 41 KDA. These properties help them to survive in the acidic rumen environment. The study provides the characteristics of secondary structures, indicating that cellulase is composed mostly of random coil followed by alpha helix and extended strand. This protein showed two functional motifs belonging to the glycoside hydrolase family. The structure evaluation and 3D alignment show that the best 3D cellulase protein model was obtained by RaptorX homology modeling program based on the 6Q1I template.

Verification of predicted 3D model by Ramachandran plot presented that most of the residues are in the allowed or favored regions of the plot. Also, the alignment analysis of this model with the template (6Q1I) supported the good quality of the predicted model, which was submitted in the PMDB database. This study gives a piece of theoretical information about the structural and functional properties of cellulase from *Ruminococcus albus* and may help for further investigations regarding the potential application of cellulase in the industry.

## Supplementary Information

**Additional file 1 **: **Fig. S1**. Multiple Sequence Alignment of cellulase sequences from different *Ruminoccocus albus* strains, generated by the use of Clustal Omega. Black shaded regions indicate similar residues.

**Additional file 2 **: **Fig. S2**. Representation of homology between the query sequence (P23660) and the selected templates from different species. Conserved residues are highlighted from blue to red colors.

**Additional file 3 **: **Fig. S3**. Prediction of subcellular localization of cellulase from *Ruminococcus albus* by TMHMM server.

**Additional file 4 **: **Fig. S4**. Representation of secondary structure alignment between the query sequence (P23660) and the selected templates from different species.

**Additional file 5 **: **Fig. S5**. Result of motif finder showing two functional motifs for the cellulase of *Ruminococcus albus.*

## Data Availability

All data generated or analyzed during this study are included in this published article.

## Abbreviations

AI: Aliphatic index
pI: Isoelectric point
II: Instability index
GRAVY: Grand average of hydropathicity
EC: Extinction coefficient
PPI: Protein–protein interaction
SARA: Subacute ruminal acidosis in dairy cows
PDB: Protein Data Bank
SPs: Signal peptides
RMSD: Root mean square deviation
