# Supplementary figures and images for: In silico Structural, Functional and Phylogenetic Analyses of cellulase from Ruminococcus albus
Source: J Genet Eng Biotechnol. 2021 Apr 19;19:58. doi: 10.1186/s43141-021-00162-x (PMC8055742; doi:10.1186/s43141-021-00162-x)

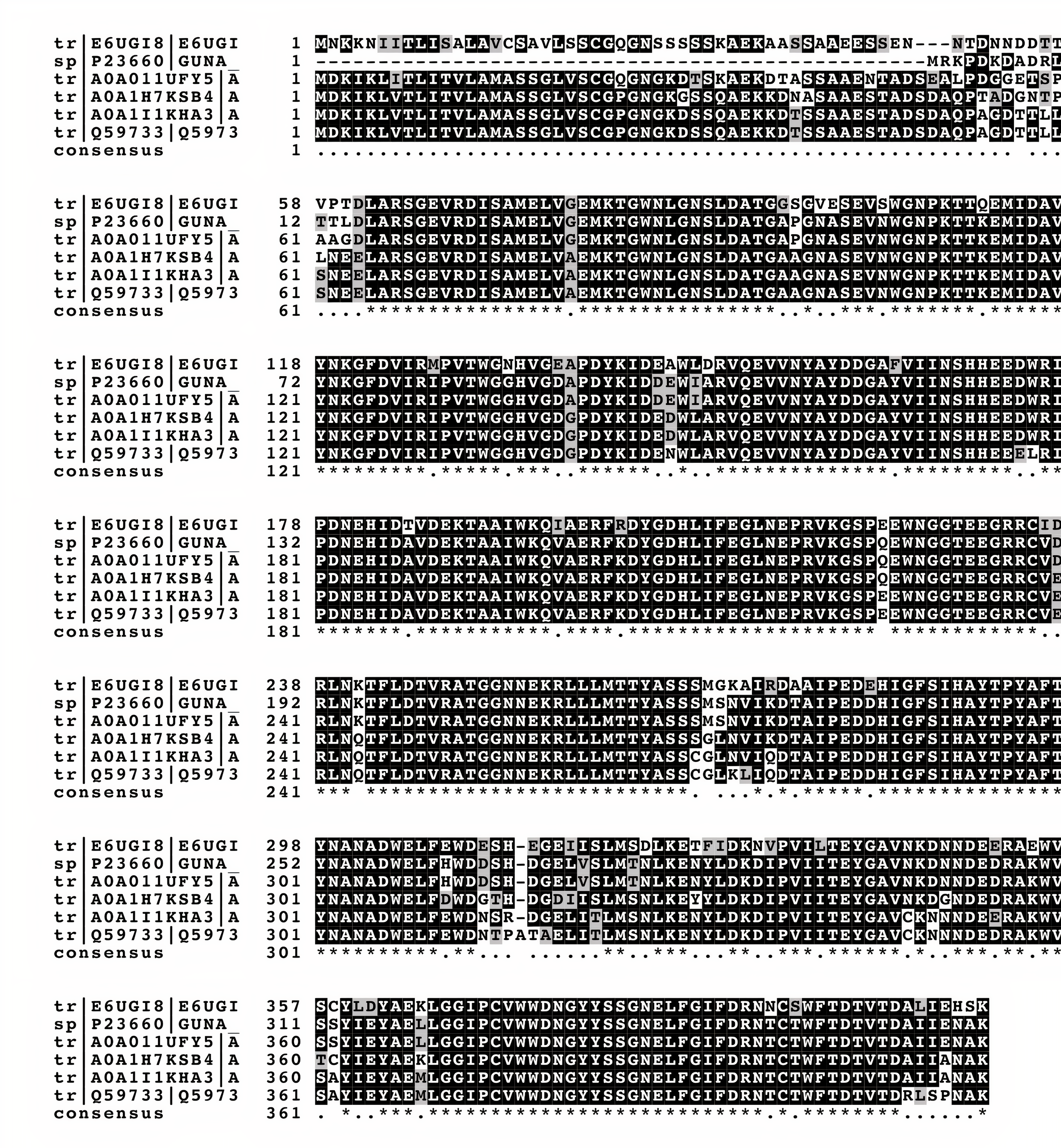

Supplement: Supplementary file 1 — Additional file 1 : Fig. S1. Multiple Sequence Alignment of cellulase sequences from different Ruminoccocus albus strains, generated by the use of Clustal Omega. Black shaded regions indicate similar residues. [file 43141_2021_162_MOESM1_ESM.png]

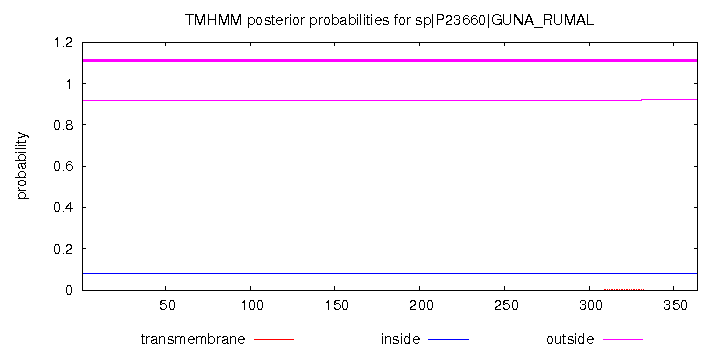

Supplement: Supplementary file 3 — Additional file 3 : Fig. S3. Prediction of subcellular localization of cellulase from Ruminococcus albus by TMHMM server. [file 43141_2021_162_MOESM3_ESM.bmp]

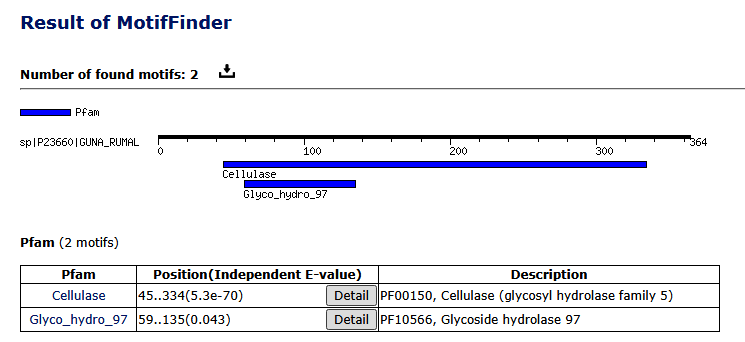

Supplement: Supplementary file 5 — Additional file 5 : Fig. S5. Result of motif finder showing two functional motifs for the cellulase of Ruminococcus albus. [file 43141_2021_162_MOESM5_ESM.png]
