# Supplementary material for: In silico Structural, Functional and Phylogenetic Analyses of cellulase from Ruminococcus albus
Source: J Genet Eng Biotechnol. 2021 Apr 19;19:58. doi: 10.1186/s43141-021-00162-x (PMC8055742; doi:10.1186/s43141-021-00162-x)
Supplement: Supplementary file 2 — Additional file 2 : Fig. S2. Representation of homology between the query sequence (P23660) and the selected templates from different species. Conserved residues are highlighted from blue to red colors. [file 43141_2021_162_MOESM2_ESM.pdf]

Results colour-coded for amino acid conservation

The current colourscheme of the alignment is for **amino acid conservation**.

The conservation scoring is performed by PRALINE. The scoring scheme works from 0 for the least conserved alignment position, up to 10 for the most conserved alignment position. The colour assignments are:

Unconserved 0 1 2 3 4 5 6 7 8 9 10 **Conserved**

```
            .........10 .........20 .........30 .........40 .........50
P23660      MRKPDKDADR LTTLDLARSG EVRDISAMEL VGEMKTGWNL GNSLDATG--
1EDG_A      MY----DASL IPNLQIPQK- NIPNNDGMNF VKGLRLGWNL GNTFDAFN--
3NDY_A      ---------- ----STAFT- GVRDVPAQQI VNEMKVGWNL GNTMD-----
6MQ4        ---------- ----SVAKTG -MRDITALEL TKDMRLGWSL GNTMDAYY--
3AYR_A      M------AHH HH-HHVDDDD KIRDISSKEL IKEMNFGWNL GNTMDAQCIE
3AYS_A      M------AHH HH-HHVDDDD KIRDISSKEL IKEMNFGWNL GNTMDAQCIE
6Q1I_A      ---------S NP-LEVQAA- --SMRSASEI VQEMGVGWNL GNTLDAKI-T
4NF7_A      ---------- -------GA- -GTDRSATQV VSDMRVGWNI GNSLDSFG-Q
6GL2_A      ---------- -HHHHHHGN- -MREIAPKEF VLDMGAGWNL GNAMDTYN--
6WQP_A      ---------S LELLEPPTQ- -MRDLTASQL LDEITIGWNL GNTLDATT-T
4IM4_A      ---------- ---------- GMRDISAIDL VKEIKIGWNL GNTLDA----
Consistency 1000000101 0101332230 1567566477 847845**99 **77*62100

            .........60 .........70 .........80 .........90 .........100
P23660      -AP----GNA SEVNWGNPKT TKEMIDAVYN KGFDVIRIPV TWGGHVGDAP
1EDG_A      -GTNITNELD YETSWSGIKT TKQMIDAIKQ KGFNTVRIPV SWHPHVS-GS
3NDY_A      -------AIG GETNWGNPMT THAMINKIKE AGFNTLRLPV TWDGHMGAAP
6MQ4        ---SAASGLA TETCWGNPKT TKAMIDKVKE AGFNTVRIPI TWAGHFGSAP
3AYR_A      YLNYEKDQTA SETCWGNPKT TEDMFKVLID NQFNVFRIPT TWSGHFGEAP
3AYS_A      YLNYEKDQTA SETCWGNPKT TEDMFKVLID NQFNVFRIPT TWSGHFGEAP
6Q1I_A      NLSYNTSPIS FETGWGNPVT TKAMIDKIKN AGFKTIRIPT TWGEHLDGN-
4NF7_A      S-YNFPYTSL NETYWGNPAT TKALIDEVAK AGFNTIRIPV SWGQYT-SGS
6GL2_A      ---------S DETAWGNPLT TKAMIDEIAK MGFKTLRLPV TWKFHIGEGP
6WQP_A      SWLPNPTPAQ SETAWGCPMT TKAMIDKVKE GGFNTVRVPV SWIDHTGSAP
4IM4_A      ---------P TETAWGNPRT TKAMIEKVRE MGFNAVRVPV TWDTHIGPAP
Consistency 0011111224 4*93*8785* *759865845 47*776*8*7 8*33956466

            .........110.........120.........130.........140.........150
P23660      DYKIDDEWIA RVQEVVNYAY DDGAYVIINS HHEEDW-RIP D---NEHIDA
1EDG_A      DYKISDVWMN RVQEVVNYCI DNKMYVILNT HHDVDK-VKG YFPSSQYMAS
3NDY_A      EYTIDQTWMK RVEEIANYAF DNDMYVIINL HHENEW-LKP F---YANEAQ
6MQ4        NYTIDSAWLS RVEEIVNYVL DNDMYAIINL HHEENT-WLV PT--YANQEV
3AYR_A      DYKIDEKWLK RVHEVVDYPY KNGAFVILNL HHET---WNH AF--SETLDT
3AYS_A      DYKIDEKWLK RVHEVVDYPY KNGAFVILNL HHET---WNH AF--SETLDT
6Q1I_A      -NKLNEEWVK RVKEVVDYCI ADDLYVILNT HHEGN--WVI PT--YAKESS
4NF7_A      DYQIPDFVMN RVKEVVDYCI VNDMYVILNS HHDINSDYCF YVPNNANKDR
6GL2_A      DYLIEANWLD KVEAIANFAL ENEMYVIINI HHDE----TW ILPTYEKADE
6WQP_A      EYQIDEAWMN RVQEVVNYVI DNDMYCILNI HHEN----DW LIPTNAQKDS
4IM4_A      DYKIDEAWLN RVEEVVNYVL DCGMYAIINL HHDN----TW IIPTYANEQR
Consistency 6859663875 9*68987945 565687*8*5 **83100232 3311464454

            .........160.........170.........180.........190.........200
P23660      VDEKTAAIWK QVAERFKDYG DHLIFEGLNE PRVKGSPQEW --------NGG
1EDG_A      SKKYITSVWA QIAARFANYD EHLIFEGMNE PRLVGHANEW WPELT--NSD
3NDY_A      VKAQLTKVWT QIANNFKKYG DHLIFETMNE PRPVGASLQW --------TGG
6MQ4        ATAQITKLWE QIATRFKDYS DYLIFEAMNE PRVVGGSAEW --------TGG
3AYR_A      AKEILEKIWS QIAEEFKDYD EHLIFEGLNE PRKNDTPVEW --------TGG
3AYS_A      AKEILEKIWS QIAEEFKDYD EHLIFEGLNA PRKNDTPVEW --------TGG
6Q1I_A      VTPKLKTLWT QISEAFKDYD DHLIFETLNQ PRLEGTPYEW --------TGG
4NF7_A      SEKYFKSIWT QIAKEFRNYD YHLVFETMNE PRLVGHGEEW WFPRNNPSND
6GL2_A      VKDELSKVWT QIANRFKTYG DYLIFETLNE PRHKGTPEEW KGG-------
6WQP_A      VNARLDAIWT QIATRFGSYD EHLIFEGMNQ PRLVGDPNEW NGG-------
4IM4_A      SKEKLVKVWE QIATRFKDYD DHLLFETMNE PREVGSPMEW M--------GG
Consistency 5554746 8*5 *9956*75*6 68*9**58*7 **3474539* 0000000344

            .........210.........220.........230.........240.........250
P23660      TEEGRRCVDR LNKTFLDTVR ATGGNNEKRL LLMTTYASSS MSNVIKDTAI
1EDG_A      VVDSINCINQ LNQDFVNTVR ATGGKNASRY LMCPGYVASP DGATNDYFRM
3NDY_A      SYENREVVNR YNLTAVNAIR ATGGNNATRY IMVPTLAASA MSTTINDLVI
6MQ4        TAENRAVINS LSLAAVNTIR ATGGNNEKRF LMVPTHAACS LTDAVNDLVI
3AYR_A      DQEGWDAVNA MNAVFLKTVR SAGGNNPKRH LMIPPYAAAC NENSFNNFIF
3AYS_A      DQEGWDAVNA MNAVFLKTVR SAGGNNPKRH LMIPPYAAAC NENSFNNFIF
6Q1I_A      TSESRDVVNK YNAAALESIR KTGGNNLSRA VMMPTY-AAS GSSTTMNDFK
4NF7_A      IREAVACIND YNQVALDAIR ATGGNNATRC VMVPGYDASI EGCMTDGFKM
```

```
6GL2_A      TQEGRDAVNQ YHQVSVDAIR ATGGNNAKRK IMVSTYAAST ASNALNDYLV
6WQP_A      NQEARQVINS YNQTFVNTVR ATGGNNAIRC LMVPTYAASC SSTTVNDFVL
4IM4_A      TYENRDVINR FNLAVVNTIR ASGGNNDKRF ILVPTNAATG LDVALNDLVI
Consistency 5495455994 584557579* 77**8*45*3 8977566963 3545465455

            .........260.........270.........280.........290.........300
P23660      PEDD-----H IGFSIHAYTP YAFT---YNA NADWELFHWD DSHDGELVSL
1EDG_A      PNDISGNNNK IIVSVHAYCP WNFAGLAMAD GGTNAWNIND SKDQSEVTWF
3NDY_A      PNN----DSK VIVSLHMYSP YFFA-----M DINGTSSWGS DYDKSSLDSE
6MQ4        PNN----DSK IIVSLHMYSP YYFA---MV- -SNSTPTWGT DSDKSSLSYE
3AYR_A      PED----DDK VIASVHAYAP YNFA---LNN -GEGAVDKFD AAGKRDLEWN
3AYS_A      PED----DDK VIASVHAYAP YNFA---LNN -GEGAVDKFD AAGKRDLEWN
6Q1I_A      VPD----DKN VIASVHAYSP YFFA---MDT SSNSVNTWGS SYDKYSLDVE
4NF7_A      PNDT--ASGR LILSVHAYIP YYFA-----L ASDTYVTRFD DNLKYDIDSF
6GL2_A      PNG----DKN VIVDVHSYFP YQFC-----L DGTDSTWGTE ADKTALLAEL
6WQP_A      PTDT--VANK LIVDIHSYSP YNFA-----L NT-SGTSSFT QSDISQLQWT
4IM4_A      PNN----DSR VIVSIHAYSP YFFA-----M DVNGTSYWGS DYDKASLTSE
Consistency 8560000546 88688*6*5* 93*8000113 2444433235 5445458433

            .........310.........320.........330.........340.........350
P23660      MTNLKENYLD KDIPVIITEY GAVNKDNNDE DRAKWVSSYI EYAELLGGIP
1EDG_A      MDNIYNKYTS RGIPVIIGEC GAVDKNN-LK TRVEYMSYYV AQAKARG-IL
3NDY_A      FDAVYNKFVK NGRAVVIGEM GSINKNN-TA ARVTHAEYYA KSAKARG-LT
6MQ4        LDAVYNKFIK NGRAVVIGEF GSIDKSN-LS SRVTHAQYYA QEATKRG-IP
3AYR_A      INLMKKRFVD QGIPMILGEY GAMNRDN-EE DRATWAEFYM EKVTAMG-VP
3AYS_A      INLMKKRFVD QGIPMILGEY GAMNRDN-EE DRATWAEFYM EKVTAMG-VP
6Q1I_A      LDSYLNTFKS KGVPVVIGQF GSINKNN-TS SRAELAEYYV TAAQKRG-IP
4NF7_A      FNDLNSKFLS RNIPVVVGET SATNRNN-TA ERVKWADYYW GRAARYSNVA
6GL2_A      DKIRDKFIVE DNRAVVMGEW GSTFSDN-PE DRLAHAEFYA RACAER-GIC
6WQP_A      LQEIYNSFGA KGIPVIIGQF GALNKNN-IN GRVLWGENYL RIAKSY-NIR
4IM4_A      LDAIYNRFVK NGRAVIIGEF GTIDKNN-LS SRVAHAEHYA REAVSRG-IA
Consistency 5535465755 5756898884 876676*035 5*644775*5 4474555094

            .........360.........370.........380.........390.........400
P23660      CVWWDNGY-Y S----SGNEL FGIFDRNTCT WFTDTVTDAI IENAK-----
1EDG_A      CILWDNNN-F S----GTGEL FGFFDRRSCQ FKFPEIIDGM VKYAFGLIN-
3NDY_A      PIWWDNGYSV A----GKAET FGIFNRSNLT WDAPEVMKAF IKGIGGSS--
6MQ4        VCWWDNGYYG P----GKDNS YALLNRSSLT WYYPEIVQAL VKGSGYTV--
3AYR_A      QIWWDNGVF- E----GTGER FGLLDRKNLK IVYPTIVAAL QKGRGLEVNV
3AYS_A      QIWWDNGVF- E----GTGER FGLLDRKNLK IVYPTIVAAL QKGRGLEVNV
6Q1I_A      CVWWDNNYAE T----NKGET FGLLNRSTLN WYFSDIKDAL IRGYK---N-
4NF7_A      MVLWDNNIYQ NNSAGSDGEC HMYIDRNSLQ WKDPEIISTI MKH-------
6GL2_A      PIWWD----N G-----NVDE FGIFNRNTLE WNYPEIAEAI VK--------
6WQP_A      CIWWDNNAFD T-----SGEN FGLLNRGTLT WQYPELLEAM MK--------
4IM4_A      VFWWDNGYYN P----GDAET YALLNRKTLS WYYPEIVQAL MRGAG-----
Consistency 377**85321 4000044583 76767*5675 6357695587 6832200110

            .........410
P23660      ----------
1EDG_A      ----------
3NDY_A      ----------
6MQ4        ----------
3AYR_A      VHAIEKETEE
3AYS_A      VHAIEKETEE
6Q1I_A      VH--------
4NF7_A      ----------
6GL2_A      ----------
6WQP_A      ----------
4IM4_A      ----------
Consistency 0000000000
```
