# Supplementary material for: In silico Structural, Functional and Phylogenetic Analyses of cellulase from Ruminococcus albus
Source: J Genet Eng Biotechnol. 2021 Apr 19;19:58. doi: 10.1186/s43141-021-00162-x (PMC8055742; doi:10.1186/s43141-021-00162-x)
Supplement: Supplementary file 4 — Additional file 4 : Fig. S4. Representation of secondary structure alignment between the query sequence (P23660) and the selected templates from different species. [file 43141_2021_162_MOESM4_ESM.pdf]

Results colour-coded for secondary structure

The current colourscheme of the alignment is for **secondary structure type**.

The 3-state (H, E C) secondary structure for each sequence is represented by a colour. If a sequence in the alignment has no colours assigned, this means that either there is no DSSP information available (if this was requested), or that no prediction was possible for that sequence (if this was requested).
The colour assignments are:

HELIX (H) **STRAND (E)** You have selected to perform secondary structure prediction using **DSSP** (Kabsch and Sander, 1983) and **PSIPRED** (Jones, 1999).

```
              .........10 .........20 .........30 .........40 .........50
(PRED) P23660 MRKPDKDADR LTTLDLARSG EVRDISAMEL VGEMKTGWNL GNSLDATG--
(PRED) 1EDG_A MY----DASL IPNLQIPQK- NIPNNDGMNF VKGLRLGWNL GNTFDAFN--
(PRED) 3NDY_A ---------- ----STAFT- GVRDVPAQQI VNEMKVGWNL GNTMD-----
(PRED) 6MQ4   ---------- ----SVAKTG -MRDITALEL TKDMRLGWSL GNTMDAYY--
(PRED) 3AYR_A M------AHH HH-HHVDDDD KIRDISSKEL IKEMNFGWNL GNTMDAQCIE
(PRED) 3AYS_A M------AHH HH-HHVDDDD KIRDISSKEL IKEMNFGWNL GNTMDAQCIE
(PRED) 6Q1I_A ---------S NP-LEVQAA- --SMRSASEI VQEMGVGWNL GNTLDAKI-T
(PRED) 4NF7_A ---------- -------GA- -GTDRSATQV VSDMRVGWNI GNSLDSFG-Q
(PRED) 6GL2_A ---------- -HHHHHHGN- -MREIAPKEF VLDMGAGWNL GNAMDTYN--
(PRED) 6WQP_A ---------S LELLEPPTQ- -MRDLTASQL LDEITIGWNL GNTLDATT-T
(PRED) 4IM4_A ---------- ---------- GMRDISAIDL VKEIKIGWNL GNTLDA----

              .........60 .........70 .........80 .........90 .........100
(PRED) P23660 -AP----GNA SEVNWGNPKT TKEMIDAVYN KGFDVIRIPV TWGGHVGDAP
(PRED) 1EDG_A -GTNITNELD YETSWSGIKT TKQMIDAIKQ KGFNTVRIPV SWHPHVS-GS
(PRED) 3NDY_A -------AIG GETNWGNPMT THAMINKIKE AGFNTLRLPV TWDGHMGAAP
(PRED) 6MQ4   ---SAASGLA TETCWGNPKT TKAMIDKVKE AGFNTVRIPI TWAGHFGSAP
(PRED) 3AYR_A YLNYEKDQTA SETCWGNPKT TEDMFKVLID NQFNVFRIPT TWSGHFGEAP
(PRED) 3AYS_A YLNYEKDQTA SETCWGNPKT TEDMFKVLID NQFNVFRIPT TWSGHFGEAP
(PRED) 6Q1I_A NLSYNTSPIS FETGWGNPVT TKAMIDKIKN AGFKTIRIPT TWGEHLDGN-
(PRED) 4NF7_A S-YNFPYTSL NETYWGNPAT TKALIDEVAK AGFNTIRIPV SWGQYT-SGS
(PRED) 6GL2_A ---------S DETAWGNPLT TKAMIDEIAK MGFKTLRLPV TWKFHIGEGP
(PRED) 6WQP_A SWLPNPTPAQ SETAWGCPMT TKAMIDKVKE GGFNTVRVPV SWIDHTGSAP
(PRED) 4IM4_A ---------P TETAWGNPRT TKAMIEKVRE MGFNAVRVPV TWDTHIGPAP

              .........110.........120.........130.........140.........150
(PRED) P23660 DYKIDDEWIA RVQEVVNYAY DDGAYVIINS HHEEDW-RIP D---NEHIDA
(PRED) 1EDG_A DYKISDVWMN RVQEVVNYCI DNKMYVILNT HHDVDK-VKG YFPSSQYMAS
(PRED) 3NDY_A EYTIDQTWMK RVEEIANYAF DNDMYVIINL HHENEW-LKP F---YANEAQ
(PRED) 6MQ4   NYTIDSAWLS RVEEIVNYVL DNDMYAIINL HHEENT-WLV PT--YANQEV
(PRED) 3AYR_A DYKIDEKWLK RVHEVVDYPY KNGAFVILNL HHET---WNH AF--SETLDT
(PRED) 3AYS_A DYKIDEKWLK RVHEVVDYPY KNGAFVILNL HHET---WNH AF--SETLDT
(PRED) 6Q1I_A -NKLNEEWVK RVKEVVDYCI ADDLYVILNT HHEGN--WVI PT--YAKESS
(PRED) 4NF7_A DYQIPDFVMN RVKEVVDYCI VNDMYVILNS HHDINSDYCF YVPNNANKDR
(PRED) 6GL2_A DYLIEANWLD KVEAIANFAL ENEMYVIINI HHDE----TW ILPTYEKADE
(PRED) 6WQP_A EYQIDEAWMN RVQEVVNYVI DNDMYCILNI HHEN----DW LIPTNAQKDS
(PRED) 4IM4_A DYKIDEAWLN RVEEVVNYVL DCGMYAIINL HHDN----TW IIPTYANEQR

              .........160.........170.........180.........190.........200
(PRED) P23660 VDEKTAAIWK QVAERFKDYG DHLIFEGLNE PRVKGSPQEW -------NGG
(PRED) 1EDG_A SKKYITSVWA QIAARFANYD EHLIFEGMNE PRLVGHANEW WPELT--NSD
(PRED) 3NDY_A VKAQLTKVWT QIANNFKKYG DHLIFETMNE PRPVGASLQW -------TGG
(PRED) 6MQ4   ATAQITKLWE QIATRFKDYS DYLIFEAMNE PRVVGGSAEW -------TGG
(PRED) 3AYR_A AKEILEKIWS QIAEEFKDYD EHLIFEGLNE PRKNDTPVEW -------TGG
(PRED) 3AYS_A AKEILEKIWS QIAEEFKDYD EHLIFEGLNA PRKNDTPVEW -------TGG
(PRED) 6Q1I_A VTPKLKTLWT QISEAFKDYD DHLIFETLNQ PRLEGTPYEW -------TGG
(PRED) 4NF7_A SEKYFKSIWT QIAKEFRNYD YHLVFETMNE PRLVGHGEEW WFPRNNPSND
(PRED) 6GL2_A VKDELSKVWT QIANRFKTYG DYLIFETLNE PRHKGTPEEW KGG-------
(PRED) 6WQP_A VNARLDAIWT QIATRFGSYD EHLIFEGMNQ PRLVGDPNEW NGG-------
(PRED) 4IM4_A SKEKLVKVWE QIATRFKDYD DHLLFETMNE PREVGSPMEW M-------GG

              .........210.........220.........230.........240.........250
(PRED) P23660 TEEGRRCVDR LNKTFLDTVR ATGGNNEKRL LLMTTYASSS MSNVIKDTAI
(PRED) 1EDG_A VVDSINCINQ LNQDFVNTVR ATGGKNASRY LMCPGYVASP DGATNDYFRM
(PRED) 3NDY_A SYENREVVNR YNLTAVNAIR ATGGNNATRY IMVPTLAASA MSTTINDLVI
(PRED) 6MQ4   TAENRAVINS LSLAAVNTIR ATGGNNEKRF LMVPTHAACS LTDAVNDLVI
(PRED) 3AYR_A DQEGWDAVNA MNAVFLKTVR SAGGNNPKRH LMIPPYAAAC NENSFNNFIF
(PRED) 3AYS_A DQEGWDAVNA MNAVFLKTVR SAGGNNPKRH LMIPPYAAAC NENSFNNFIF
(PRED) 6Q1I_A TSESRDVVNK YNAAALESIR KTGGNNLSRA VMMPTY-AAS GSSTTMNDFK
(PRED) 4NF7_A IREAVACIND YNQVALDAIR ATGGNNATRC VMVPGYDASI EGCMTDGFKM
(PRED) 6GL2_A TQEGRDAVNQ YHQVSVDAIR ATGGNNAKRK IMVSTYAAST ASNALNDYLV
(PRED) 6WQP_A NQEARQVINS YNQTFVNTVR ATGGNNAIRC LMVPTYAASC SSTTVNDFVL
(PRED) 4IM4_A TYENRDVINR FNLAVVNTIR ASGGNNDKRF ILVPTNAATG LDVALNDLVI
```

```
                 . . . . . . . . . 260. . . . . . . . . 270. . . . . . . . . 280. . . . . . . . . 290. . . . . . . . . 300
(PRED)  P23660  PEDD-----H IGFSIHAYTP YAFT---YNA NADWELFHWD DSHDGELVSL
(PRED)  1EDG_A  PNDISGNNNK IIVSVHAYCP WNFAGLAMAD GGTNAWNIND SKDQSEVTWF
(PRED)  3NDY_A  PNN----DSK VIVSLHMYSP YFFA-----M DINGTSSWGS DYDKSSLDSE
(PRED)  6MQ4    PNN----DSK IIVSLHMYSP YYFA---MV- -SNSTPTWGT DSDKSSLSYE
(PRED)  3AYR_A  PED----DDK VIASVHAYAP YNFA---LNN -GEGAVDKFD AAGKRDLEWN
(PRED)  3AYS_A  PED----DDK VIASVHAYAP YNFA---LNN -GEGAVDKFD AAGKRDLEWN
(PRED)  6Q1I_A  VPD----DKN VIASVHAYSP YFFA---MDT SSNSVNTWGS SYDKYSLDVE
(PRED)  4NF7_A  PNDT--ASGR LILSVHAYIP YYFA-----L ASDTYVTRFD DNLKYDIDSF
(PRED)  6GL2_A  PNG----DKN VIVSVHSYFP YQFC-----L DGTDSTWGTE ADKTALLAEL
(PRED)  6WQP_A  PTDT--VANK LIVDIHSYSP YNFA-----L NT-SGTSSFT QSDISQLQWT
(PRED)  4IM4_A  PNN----DSR VIVSIHAYSP YFFA-----M DVNGTSYWGS DYDKASLTSE

                 . . . . . . . . . 310. . . . . . . . . 320. . . . . . . . . 330. . . . . . . . . 340. . . . . . . . . 350
(PRED)  P23660  MTNLKENYLD KDIPVIITEY GAVNKDNNDE DRAKWVSSYI EYAELLGGIP
(PRED)  1EDG_A  MDNIYNKYTS RGIPVIIGEC GAVDKNN-LK TRVEYMSYYV AQAKARG-IL
(PRED)  3NDY_A  FDAVYNKFVK NGRAVVIGEM GSINKNN-TA ARVTHAEYYA KSAKARG-LT
(PRED)  6MQ4    LDAVYNKFIK NGRAVVIGEF GSIDKSN-LS SRVTHAQYYA QEATKRG-IP
(PRED)  3AYR_A  INLMKKRFVD QGIPMILGEY GAMNRDN-EE DRATWAEFYM EKVTAMG-VP
(PRED)  3AYS_A  INLMKKRFVD QGIPMILGEY GAMNRDN-EE DRATWAEFYM EKVTAMG-VP
(PRED)  6Q1I_A  LDSYLNTFKS KGVPVVIGQF GSINKNN-TS SRAELAEYYV TAAQKRG-IP
(PRED)  4NF7_A  FNDLNSKFLS RNIPVVVGET SATNRNN-TA ERVKWADYYW GRAARYSNVA
(PRED)  6GL2_A  DKIRDKFIVE DNRAVVMGEW GSTFSDN-PE DRLAHAEFYA RACAER-GIC
(PRED)  6WQP_A  LQEIYNSFGA KGIPVIIGQF GALNKNN-IN GRVLWGENYL RIAKSY-NIR
(PRED)  4IM4_A  LDAIYNRFVK NGRAVIIGEF GTIDKNN-LS SRVAHAEHYA REAVSRG-IA

                 . . . . . . . . . 360. . . . . . . . . 370. . . . . . . . . 380. . . . . . . . . 390. . . . . . . . . 400
(PRED)  P23660  CVWWDNGY-Y S----SGNEL FGIFDRNTCT WFTDTVTDAI IENAK-----
(PRED)  1EDG_A  CILWDNNN-F S----GTGEL FGFFDRRSCQ FKFPEIIDGM VKYAFGLIN-
(PRED)  3NDY_A  PIWWDNGYSV A----GKAET FGIFNRSNLT WDAPEVMKAF IKGIGGSS--
(PRED)  6MQ4    VCWWDNGYYG P----GKDNS YALLNRSSLT WYYPEIVQAL VKGSGYTV--
(PRED)  3AYR_A  QIWWDNGVF- E----GTGER FGLLDRKNLK IVYPTIVAAL QKGRGLEVNV
(PRED)  3AYS_A  QIWWDNGVF- E----GTGER FGLLDRKNLK IVYPTIVAAL QKGRGLEVNV
(PRED)  6Q1I_A  CVWWDNNYAE T----NKGET FGLLNRSTLN WYFSDIKDAL IRGYK---N-
(PRED)  4NF7_A  MVLWDNNIYQ NNSAGSDGEC HMYIDRNSLQ WKDPEIISTI MKH-------
(PRED)  6GL2_A  PIWWD----N G-----NVDE FGIFNRNTLE WNYPEIAEAI VK--------
(PRED)  6WQP_A  CIWWDNNAFD T-----SGEN FGLLNRGTLT WQYPELLEAM MK--------
(PRED)  4IM4_A  VFWWDNGYYN P----GDAET YALLNRKTLS WYYPEIVQAL MRGAG-----

                 . . . . . . . . . 410
(PRED)  P23660  ----------
(PRED)  1EDG_A  ----------
(PRED)  3NDY_A  ----------
(PRED)  6MQ4    ----------
(PRED)  3AYR_A  VHAIEKETEE
(PRED)  3AYS_A  VHAIEKETEE
(PRED)  6Q1I_A  VH--------
(PRED)  4NF7_A  ----------
(PRED)  6GL2_A  ----------
(PRED)  6WQP_A  ----------
(PRED)  4IM4_A  ----------
```
